# Immunogenicity of an AAV-Based COVID-19 Vaccine in Murine Models of Obesity and Aging

**DOI:** 10.3390/v14040820

**Published:** 2022-04-15

**Authors:** Dawid Maciorowski, Cheikh Diop, Urja Bhatt, Reynette Estelien, Dan Li, Ruchi Chauhan, Luk H. Vandenberghe, Nerea Zabaleta

**Affiliations:** 1 Grousbeck Gene Therapy Center, Schepens Eye Research Institute, Mass Eye and Ear, Boston, MA 02114, USA; maciorowski@wisc.edu (D.M.); cheikh_diop@meei.harvard.edu (C.D.); urja_bhatt@meei.harvard.edu (U.B.); reynette_estelien@meei.harvard.edu (R.E.); dan_li@meei.harvard.edu (D.L.); ruchi.chauhan.rcs@gmail.com (R.C.); 2 Department of Ophthalmology, Ocular Genomics Institute, Harvard Medical School, Boston, MA 02114, USA; 3 The Broad Institute of Harvard and MIT, Cambridge, MA 02142, USA; 4 Harvard Stem Cell Institute, Harvard University, Cambridge, MA 02138, USA

**Keywords:** SARS-CoV-2, COVID-19, vaccine, genetic vaccine, adeno-associated virus, AAV, obesity, aging, animal model

## Abstract

The SARS-CoV-2 pandemic has had a disastrous impact on global health. Although some vaccine candidates have been effective in combating SARS-CoV-2, logistical, economical, and sociological aspects still limit vaccine access globally. Recently, we reported on two room-temperature stable AAV-based COVID-19 vaccines that induced potent and protective immunogenicity following a single injection in murine and primate models. Obesity and old age are associated with increased mortality in COVID-19, as well as reduced immunogenicity and efficacy of vaccines. Here, we investigated the effectiveness of the AAVCOVID vaccine candidates in murine models of obesity and aging. Results demonstrate that obesity did not significantly alter the immunogenicity of either vaccine candidate. In aged mice, vaccine immunogenicity was impaired. These results suggest that AAV-based vaccines may have limitations in older populations and may be equally applicable in obese and non-obese populations.

## 1. Introduction

SARS-CoV-2, the causative agent of COVID-19, was first reported in December 2019 in the Wuhan region of China [1]. The pandemic has affected every nation, causing severe mortality, morbidity, and economic distress. The magnitude of this pandemic initiated the development of numerous vaccine candidates across various technology platforms [2]. We recently published our work on the design and immunogenicity of two recombinant adeno-associated virus (AAV)-based COVID-19 vaccine candidates (AAVCOVID) in murine and primate models [3]. The two vaccine candidates were named AC1 and AC3, where AC1 encodes a full-length membrane-anchored prefusion-stabilized S protein and AC3 expresses a secreted S1 subunit of SARS-CoV-2. We showed that the AAVCOVID candidates elicited strong B and T cell immunogenic responses following a single intramuscular injection in rodents and non-human primates (NHP), which resulted in near-sterilizing protection in a NHP SARS-CoV-2 challenge model. This, along with the thermostability and manufacturing potential, led to the conclusion that AAVCOVID can serve as a promising vaccine candidate.

Obesity is a disease characterized by the accumulation of fat tissue which can lead to several serious complications [4]. The prevalence of obesity is much greater in countries in the western hemisphere, where there is more stationary lifestyles and highly processed foods with little nutritional value [4]. With COVID-19, a respiratory disease, obesity also can take its toll. Most hospitalized patients with COVID-19 present with respiratory distress [5]. With regards to respiratory diseases, obese individuals are more likely to be hospitalized than lean individuals [6]. This is thought to occur because obesity is associated with several pathologies, such as a decrease in diaphragmatic excursion and functional capacity [4]. Several groups found that obese individuals infected with SARS-CoV-2 were more likely to develop serious COVID-19 pathology [7]. This connection is not unique to COVID-19, as the relationship between obesity and the severity of viral infection was reported during the 2009 influenza pandemic [6]. Aside from the effects of obesity on disease morbidity, it also has profound effects on vaccine efficacy. The relationship between vaccine efficacy and obesity has been pondered since a breakout study in 1985 during a regular administration of hepatitis B vaccines in hospital workers [8]. The study found that vaccinated hospital staff that were obese had a significant decline in protective hepatitis B antibodies. Additionally, other studies have shown similar consequences of obesity on vaccine induction in other vaccines, such as rabies, tetanus, hepatitis A, influenza, and more [9,10,11]. With the recent influenza pandemic of 2009, more studies on obesity and vaccine immune induction were performed, with some in controlled murine models of obesity. Several of these murine models of obesity showcased that obesity led to poor pathogen-immune responses against influenza infection [12,13]. Studies on vaccination and obesity against SARS-CoV-2 have been especially limited, but a recent study found that obesity may dampen the effect of COVID-19 vaccine immunogenicity [14]. However, this is in contrast to several post hoc analyses of human data with COVID-19 vaccination which did not find a major difference in efficacy in participants with obesity versus those without [15]. Even with the limited data available, The Obesity Society, a leading scientific society for obesity, issued a statement reaffirming the efficacy of the current COVID-19 vaccines for people suffering from obesity [16]. They did include limitations in their interpretations of the studies due to a lack of information in terms of the relative efficacy in the different subtypes of obesity and a limitation of statistical testing specific to the relation of vaccine efficacy in terms of obesity. More follow-up studies are needed to fully understand the effect of obesity on COVID-19 vaccination efficacy.

Older populations have increased over the past several decades due to factors such as innovations in science and better medical care. One of the most considerable and well-studied consequences of aging is immunological senescence. This is coupled with the enhanced propensity to autoimmune conditions that impact adaptive immune responses and contribute to a chronic state of inflammation [17]. There are a handful of vaccines that are recommended for individuals over the age of 60, and all of them have some reported diminished response in elderly people. In particular, the vaccines against influenza and pneumococcal disease were found to not induce protective immunity in large proportions of the elderly population, though it was seemingly able to mitigate disease severity to some degree [18,19]. The summation of these studies suggests that vaccines may not be able to protect the elderly population as effectively as needed. There have been attempts to improve vaccine immunogenicity in elderly populations. These include the use of adjuvants, boosters, and higher doses. As COVID-19 vaccinations continue to ramp up across the world, more studies which assess the immunogenicity and possible efficacy of protecting elderly populations against COVID-19 have been conducted. Early vaccination data were promising in clinical trials that included elderly people. For example, in late 2020, the vaccine candidate mRNA-1273 was shown to be highly protective [20]. Other studies assessing BNT162b2 or ChAdOx1nCoV-19 vaccines on elderly populations also found there was a significant risk reduction from COVID-19 lethality when vaccinated [21]. On the contrary, other studies showcased that older age had a serious detrimental effect on the immune response to these vaccines, suggesting that they may be less efficacious in aging populations [22]. The conclusion from these studies remains unclear in terms of protection for the elderly because there is currently no known baseline of protection against SARS-CoV-2.

In light of the impact of COVID-19 on elderly and obese populations, it is of absolute importance to further investigate and enhance the efficacy of COVID-19 vaccines in these populations. Thus, for our studies, we evaluated the immune response of our experimental AAV-based COVID-19 vaccines against murine models of obesity and aging, with the prior understanding of the real-life limitations of murine models.

## 2. Materials and Methods

*AAVCOVID Vaccine Candidates:* Our study utilized two AAV-based vaccines: AAVCOVID-19-1 (AC1) and AAVCOVID-19-3 (AC3) (GenBank MW408785 and MW408786). AC1 is an AAVrh32.33 vector that expresses the codon-optimized, prefusion-stabilized (furin cleavage site mutated to G682SAS685 and P986P987 substitutions) full-length SARS-CoV-2 spike protein under the direction of a SV40 promoter. AC1 carries a short SV40 polyadenylation signal (poly-A). AC3 is an AAVrh32.33 that carries the secreted S1 subunit of the SARS-CoV-2 spike with the tissue plasminogen activator signal peptide (tPA-SP). This expression is directed by a CMV promoter. AC3 has two more regulatory elements: a woodchuck hepatitis virus posttranscriptional regulatory element (WPRE) and a bovine growth hormone polyadenylation signal (poly-A). 

*Production of AAVCOVID Candidates:* Research-grade, high-titer vectors were produced, purified, and titrated by the MEEI/SERI Gene Transfer Vector Core (https://www.vdb-lab.org/vector-core/ (accessed on 14 April 2022)). Small-scale vector preparations were generated by triple transfection of AC1 or AC3 ITR-flanked transgene, pKan2/rh32.33 (AAV2 rep and AAVrh32.33 capsid construct), and pALD-X80 adenoviral helper plasmid in a 1:1:2 ratio using polyethylenimine or PEI (Polysciences, Cat #24765-2). Ten-layer HYPERFlasks were used to seed HEK 293 cells and DNA transfected a PEI-Max/DNA ratio of 1.375:1 (*v*/*w*). After 3 days of incubation, vectors were harvested from the HYPERFlasks using benzonase (EMD Millipore, catalog no.1016970010) to undergo DNA/RNA degradation. One day after harvesting, the vectors were concentrated by tangential flow filtration (TFF) and purified by iodixanol gradient ultracentrifugation as previously described [23]. Vector titers were quantified by ddPCR as previously described [24]. 

*Murine Studies:* All mouse studies were designed and performed in compliance with the Schepens Eye Research Institute IACUC. C57BL/6 or C57BL/6 diet-induced obese (DIO) animals were intramuscularly (right gastrocnemius muscle) injected at 10^10^ gc/mouse or 10^11^ gc/mouse. C57BL/6 animals were kept in standard diet and C57BL/6 DIO were fed a high-fat diet (Research Diets, Cat#D12492i). These mice were injected intramuscularly in the gastrocnemius muscle, bled at regular intervals, and analyzed for SARS-CoV-2 RBD IgG and pseudo-virus neutralization responses in the serum. Serum samples were obtained by submandibular bleeds for humoral immune response analyses. At necropsy, spleens were harvested for extraction of splenocytes and analysis of cellular responses to Spike. 

*SARS-CoV-2 Spike-binding antibody detection ELISA:* Nunc MaxiSorp^TM^ high protein-binding capacity 96 well plates (Thermo Fisher Scientific, Cat# 44-2404-21) were coated with 1 μg/mL SARS-CoV-2 RBD diluted in phosphate-buffered saline (PBS) and stored overnight at 4 °C. The next day, plates were washed with PBS-Tween 20 0.05% (Sigma, Cat# P2287-100ML) using the Biotek 405 TS microplate washer. Each plate was washed five times with 200 μL of wash buffer and then tapped dry before the next step. Following the first wash, 200 μL of casein blocker in PBS (Thermo Fisher Scientific, Cat# 37528) was added to each well and then incubated for 2 h at RT. After blocking, serum samples were serially diluted in blocking solution, starting with a 1:100 dilution. After an hour of incubation, the plates were washed and 100 μL of secondary Peroxidase AffiniPure Rabbit Anti-Mouse IgG (Jackson ImmunoResearch, Cat# 315-035-045, RRID: AB_2340066) antibody (with a 1:1000 dilution) was added in blocking solution to each well. After incubating this for one hour at room temperature, the plates were washed and subsequently developed for 3.5 min with 100 μL of Seracare SureBlue Reserve^TM^ TMB Microwell Peroxidase Substrate solution (SeraCare, Cat# 53-00-03). The reaction was stopped with 100 μL of Seracare KPL TMB Stop solution (SeraCare, Cat# 50-85-06). The optical density (OD) at 450 nm was measured using the Biotek Synergy H1 plate reader. The titer was the reciprocal of the highest dilution with absorbance values higher than four times the average of the negative control wells. 

*Lenti-SARS2 pseudovirus production and titration:* Lenti-SARS2 was produced based on a published protocol [25]. Then, 50% of confluent HEK293T cells were seeded 24 h prior to the transfection in 15 cm plates. The next day, 18 μg of psPAX2, 9 μg of pCMV-SARS2-RRAR_ILR_gp41, and 29 μg of pCMV-Lenti-Luc plasmids were mixed in 3.6 mL of Opti-MEMTM I Reduced Serum Media (Gibco, Cat# 31985070). This was then added to 144 μL of PEI Max 40K (1 mg/mL, pH: 6.9–7.1) and the entire solution was mixed thoroughly. The mixture was incubated for 20 min at room temperature. The media on the cells were aspirated and serum-free DMEM was added to cells. After 20 min, the DNA-PEI mixture was added dropwise to the plate. This was then incubated overnight at 37 °C with 5% CO_2_. The next day, the media were replaced with DMEM with 10% FBS. After 48 h, the media were collected in a 50 mL conical and centrifuged at 4000 rpm at 4 °C for 5 min to remove cell debris. The supernatant was collected and filtered through 0.45 μm filter, aliquoted and stored at −80 °C. 

For the titration of the pseudovirus, HEK293T cells expressing ACE2 were seeded at 1.5 × 104 cells/well in poly-L-Lysine (0.01%) precoated 96-well black plates (Thermo Fisher Scientific, Cat# 3904) one day before titration. On the next day, the media were changed to 50 μL of DMEM + 10%FBS containing filtered hexadimethrine bromide at a final concentration of 10 μg/mL. Two-fold serial dilutions (up-to 15 dilutions) of the viral stocks (50μL) were added to the plate in 6 replicates each and then incubated for 48 h. After incubation, cells were lysed with reporter lysis buffer (Promega, Cat# E4030). These plates were frozen at −80 °C for 60 min to enhance lysis. Thereafter, they were thawed at 37 °C for 20 min before starting a luciferase readout. For the luciferase substrate buffer, the following reagents were mixed: Tris-HCl buffer at 0.5 M, ATP at 0.3 mM, MgCl_2_ at 10 mM, PierceTM Firefly Signal Enhancer (Thermo Fisher Scientific, Cat#16180), and 150μg/mL of D-luciferin (PerkinElmer, Cat# 122799). A Biotek Synergy H1 plate reader was used for luminescence readout. For the pseudo-virus neutralization assay, a final dilution of the virus stock targeting relative luminescence units (RLU) of 1800–1100 was used which was approximately 200-fold higher than background signal obtained in untreated cells. 

*Pseudovirus Neutralization Assay:* HEK293T cells expressing ACE2 were seeded at 1.5 × 10^4^ cells/well in poly-L-Lysine (0.01%) coated 96-well black plates. The following day, 50 μL of DMEM + 10% FBS media containing hexadimethrine bromide (final concentration 10 μg/mL) was added to the cells. Serum samples were then heat-inactivated for 1 h at 56 °C. Serum samples were then serially diluted (2-fold) for 10 dilutions in DMEM with 10% FBS with initial dilutions of 1:40 for the mouse serum and 1:10 dilution for NHP serum. Afterwards, Lenti-SARS2 pseudo-virus was added to each dilution and incubated at 37 °C for 45 min. The serum and virus mixture was added to the cells and incubated at 37 °C with 5% CO_2_ for 48 h. An anti-SARS-CoV-2 spike monoclonal neutralizing antibody (GenScript, Cat# 6D11F2) was used as a positive control. Cells without serum along with the virus were used as a negative control. After 48 h, cells were then lysed, and luciferase levels were measured as described above. Neutralizing antibody titers or 50% inhibitory concentration in the serum sample (EC50 or ID50) were calculated as the reciprocal of the highest dilution, showing less RLU signal than half of the average RLU (maximum infectivity) of the virus control group (cells + virus, without serum). 

*IFN-γ assay in murine splenocytes:* Splenocytes were obtained by grinding murine spleens with 100 μm cell strainers. This was then followed by treatment with ammonium chloride–potassium (ACK) lysis buffer (Gibco) to lyse the red blood cells. The isolated cells were then suspended in complete RPMI-1640 medium (Gibco), supplemented with 10% FBS, and then counted for the following experiments. 

IFN-γ ELISPOT for mice was measured using the BD Bioscience IFN-γ ELISPOT set (Cat# BDB551083). Next, 96-well plates were pre-coated with 10 ug/mL of anti-mouse IFN-γ ELISPOT capture antibody (BD Biosciences Cat# BDB551083) at 4 °C overnight, and then blocked with complete RPMI-1640 medium for 2 h at 37 °C. One million freshly isolated splenocytes were seeded into the precoated plates and stimulated with S1 and S2 peptides pools (GenScript) with a final concentration of 1 μg/mL of each peptide diluted in RPMI-1640, supplemented with 10% FBS and incubated for 48 h at 37 °C with 5% CO_2_. Each peptide pool consisted of 15-mer peptides overlapping by 10 amino acids, spanning the entire SARS-CoV-2 spike protein S1 or S2 subunits. Control wells contained 1 × 10^6^ cells stimulated with DMSO diluted in RPMI-1640 supplemented with 10% FBS (negative control) or 2 μg/mL concanavalin A (positive control). Plates were then washed and incubated with IFN-γ ELISPOT detection antibody (BD Biosciences Cat# BDB551083) at room temperature for 2 h. This was followed by the addition of streptavidin-HRP (dilution 1:1000 BD Biosciences Cat# BDB551083) for one hour. After washing, 100 μL/well of the kit’s final substrate solution was added and developed for 15–30 min until distinct spots emerged. The cytokine-secreting cell spots were imaged and counted on the AID EliSpot reader (Autoimmun Diagnostika GmbH). After this, 1 × 10^6^ freshly isolated splenocytes were seeded into 96-well plates and stimulated with 1 μg/mL of peptides from S1 and S2 pool, as described previously, at 37 °C for 48 h. Then, the supernatants were collected and cytokine levels were measured with a Luminex cytokine assay by SBH Sciences. 

*Graphs and statistical analysis:* GraphPad Prism 8 was used for graph preparation and statistical analysis. Data were represented as mean ± standard deviation (SD). Groups were compared between them by one-way ANOVA and Tukey’s tests in studies with more than two groups and *n* ≥ 10, and the Kruskal–Wallis test and Dunn’s test were used if *n* < 10. Two groups were compared between them using Student’s *t*-test (if *n* ≥ 10).

## 3. Results

### 3.1. The Immunogenicity of AAVCOVID Is Not Significantly Influenced by Obesity in a Murine Model

A diet-induced obesity (DIO) C57BL/6 mouse model was used to study vaccine efficacy in inducing SARS-CoV-2 RBD-specific antibodies in obese animals. Twelve-week-old C57BL/6 and C57BL/6 DIO (*n* = 10) mice were vaccinated with 10^10^ and 10^11^ gc of AC1 and AC3. These candidates were previously reported [3]. Briefly, AC1 expresses the prefusion-stabilized full-length Wuhan spike under the control of an SV40 promoter, and AC3 expresses a secreted monomeric S1 subunit controlled by a CMV promoter. At the time of vaccination, there was a clear average difference in weight between the wild-type and DIO groups, suggesting that the DIO group developed obesity through the uptake of a high fat diet (Figure S1).

**Figure 1:**
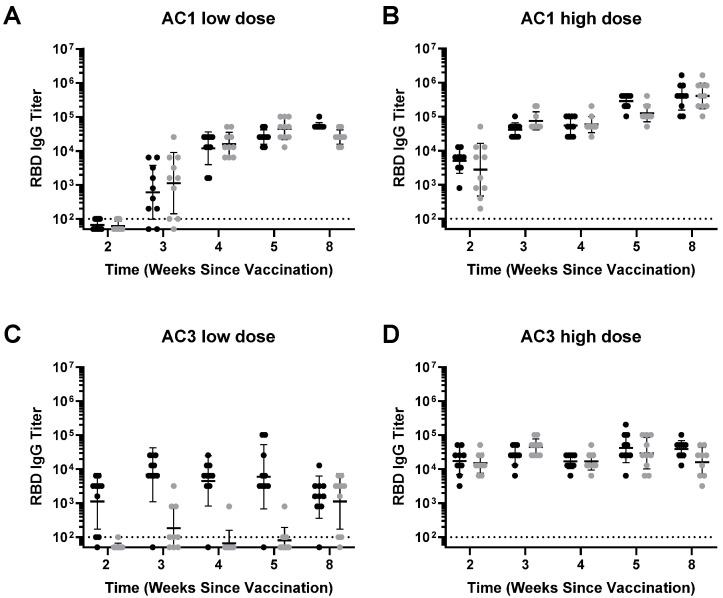
AAVCOVID vaccines elicit a high humoral response in an obese mouse model. RBD-binding antibody titer in lean and DIO animals vaccinated with low (**A**) and high (**B**) AC1 doses and low (**C**) and high (**D**) AC3 doses. Mean ± SD. The Student’s *t*-test statistical test was used to compare both groups.

**Figure 2:**
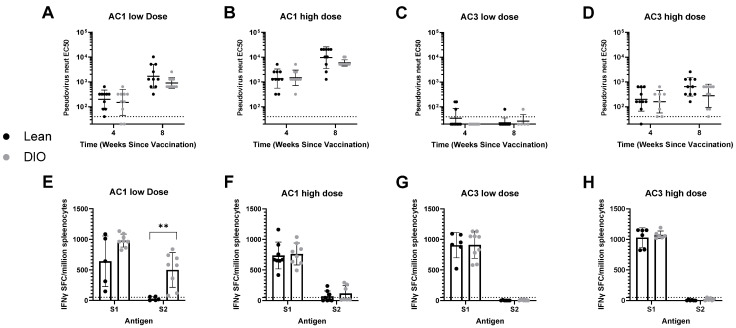
Neutralizing antibody and T-cell immuno-characterization in AAVCOVID-immunized DIO and lean mice. (**A**–**D**) Pseudo-virus neutralization (EC50) in DIO and lean animals vaccinated with low (**A**) and high (**B**) AC1 doses and low (**C**) and high (**D**) AC3 doses. (**E**–**H**) IFN-γ ELISPOT analysis (spot forming units per million splenocytes) in DIO and lean animals vaccinated with low (**E**) and high (**F**) AC1 doses and low (**G**) and high (**H**) AC3 doses. Data are represented as mean ± SD. The Student’s *t*-test statistical test was used to compare both groups. ** *p* < 0.01. Dotted lines indicate a lower limit of detection.

Upon vaccination with AC1, we found that the IgG RBD-binding and neutralizing antibody levels were indistinguishable between lean and obese groups (Figure 1A,B and Figure 2A,B). The same result was found upon immunization of AC3 at a high dose (Figure 1D and Figure 2D). When AC3 was administered at a low dose, reduction in binding antibody response was observed in the DIO mice, in comparison to the lean mice (Figure 1C). However, most of the DIO mice in the AC3 low-dose cohort developed binding antibodies on week 8, possibly indicating a delayed response. Most of the animals vaccinated with AC3 at a low dose failed to develop spike neutralizing antibody titers above the detection limit of the assay (1:40 dilution) (Figure 2C), independent of the diet. Neutralization curves are shown in Figure S2.

To further assess the cellular immune response to SARS-CoV-2 spike protein elicited by AAVCOVID vaccines in DIO mouse models, we conducted IFN-γ ELISPOT assays in splenocytes extracted from the vaccinated animals 8 weeks after immunization (Figure 2E–H). AC3-injected animals showed responses specific to the S1 subunit of the spike protein, and the high dose of AC3 resulted in a slightly higher spot count than the lower dose (Figure 2G,H). In both low and high doses of AC3, there was no difference in the response between lean and DIO groups. In AC1, we had responses in both S1 and S2 subunits, with a lower response in S2, as previously reported [3]. In high-dose AC1, there was no difference in response between lean and DIO groups (Figure 2F). In low-dose AC1, the DIO group shows a trend to a greater response against S1 peptides, but these data are inconclusive due to the variability in response to the lean group, as well as a limited sample number (Figure 2E). However, the DIO animals vaccinated with low doses of AC1 showed significantly higher responses against the S2 peptides compared to lean mice (Figure 2E). Overall, our studies suggest that obesity had no significant effect on the immunogenicity of AAVCOVID.

### 3.2. The Immunogenicity of AAVCOVID Is Influenced by Age in a Murine Model

**Figure 3:**
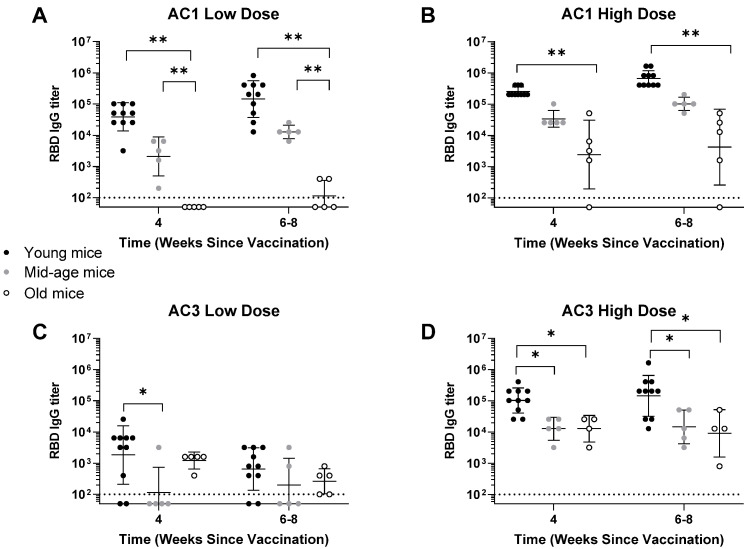
Humoral IgG characterization of AAVCOVID-immunized mice of different age groups. SARS-CoV-2 IgG ELISA was performed on varied age groups of mice vaccinated with low (**A**) and high (**B**) AC1 doses and low (**C**) and high (**D**) AC3 doses. Dotted lines indicate a lower limit of detection. Data are represented as mean ± SD. A one-way ANOVA statistical test was used to compare groups. * *p* < 0.05, ** *p* < 0.01. Dotted lines indicate a lower limit of detection.

We modeled aging in a murine C57BL/6 model by immunizing 6-week-old (young), 18-week-old (mid-aged), and 2-year-old (old) male mice with AAVCOVID vaccine candidates at low and high doses. These subjects were injected intramuscularly, bled at regular intervals, and analyzed for SARS-CoV-2 RBD IgG and pseudo-virus neutralization responses in the serum. At early timepoints after vaccination (4 to 8 weeks), the humoral response was found to be affected by the age of the mice at the time of vaccination. Animals receiving AC1 showed a downwards trend of RBD-binding antibodies in older animals (Figure 3A,B), which was significant at low doses (Figure 3A). This trend was present in animals vaccinated with AC3; however, the differences between ages were smaller and no evident difference was found between mid-age and old animals (Figure 3C,D).

**Figure 4:**
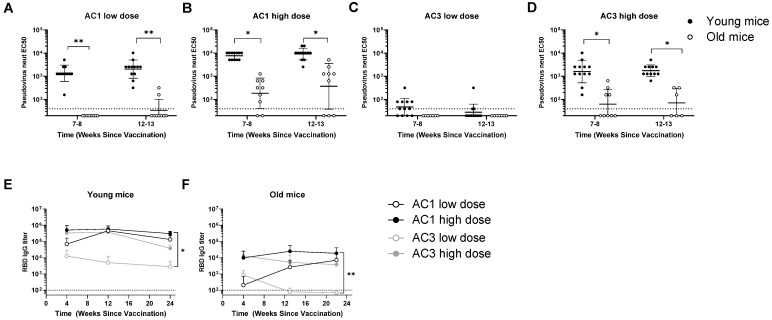
Humoral characterization of AAVCOVID-immunized mice of varied age groups. (**A**–**D**) Pseudo-virus neutralization (EC50) for young, mid-age, and old mice vaccinated with low (**A**) and high (**B**) AC1 doses and low (**C**) and high (**D**) AC3 doses. Data are represented as mean ± SD. Dotted lines indicate a lower limit of detection. The Student’s *t*-test statistical test was used to compare groups. (**E**,**F**) RBD-binding antibody titer in young and old-aged mice. data are represented as Mean ± SD. A one-way ANOVA statistical test was used to compare both groups. * *p* < 0.05, ** *p* < 0.01.

Neutralization responses and durability of antibody responses were also evaluated in young (6-week-old) and old (2-year-old) male and female C57BL/6 animals (*n* ≥ 4 animals/sex/group) (Figure 4 and Figure S3). Neutralizing responses showed the same age differences that were observed for binding antibodies, which were significantly lower in aged mice and most presented titers lower than the detection limit of the assay (1:40) (Figure 4A–D). AC1 at a high dose, the most potent candidate, presented the most promising results in aged mice regarding neutralizing titer and seroconversion (Figure 4B). 

Binding antibody responses were measured in these animals for 5–6 months (Figure 4E,F). Animals vaccinated with AC1 presented with durable immunogenicity over time, while animals vaccinated with AC3 showed a slight waning effect. These observations were true for young and old mice, indicating that the magnitude of the response is affected by age, but not its durability.

## 4. Discussion

The SARS-CoV-2 pandemic has caused major harm to global health and economic stability. The development of several safe, efficacious, and cost-effective vaccines has been a cornerstone to lowering the burden of COVID-19. A remarkable effort has already led to the development and authorization of a handful of COVID-19 vaccines that have proven to be effective overall. Further studies on these vaccines must be performed to elucidate their efficacy on vulnerable populations, such as the elderly and the obese. In an earlier study, we reported the creation of two novel COVID-19 vaccine candidates, dually named AAVCOVID. Our current study was performed to assess AAVCOVID’s immune response in murine models of aging and obesity.

Obesity serves as a major co-morbidity with COVID-19. Aside from the several pathological outcomes of obesity, a particularly important one is the ability of adipose tissue to induce pro-inflammatory cytokines [26]. It is suspected that in obese individuals, the summation of the increased amount of adipose tissue leads to a state of chronic mild inflammation. It is hypothesized that this chronic inflammation may dampen the immune responses that are induced by vaccines, thus potentially rendering them less effective. We found that both AAVCOVID candidates, AC1 and AC3, induced a potent antibody and t-cell response in the DIO models, which was no different than the comparative lean group. The utilization of DIO mouse models in assessing vaccine efficacy has been a relatively new concept, and it is still not clear how well DIO models can represent obesity in humans. Several studies have used DIO models to assess vaccine efficacy, though the interpretations of the results have been mixed [12,13,27]. We will be able to better understand the sensitivity of the DIO model as these studies are followed up with challenge trials, and then human trials that incorporate an obese population with a fat heavy diet. Obesity seems to decrease vaccine efficacy in the studies published by others [12,13]. In the case of AAVCOVID, particularly with AC3, we see a similar scenario, as the lower dose in DIO mice indicates a reduction; however, AC1′s immunogenicity is not affected in DIO mice in the conditions tested. 

Infections are major causes of morbidity and mortality in the elderly population. This is the case in COVID-19 where severity of the disease increases with age [28]. A recent study involving the Ad26.COV2.S COVID-19 vaccine looked into the immunogenicity and efficacy of vaccines in adult (3.3–5.0 years old) and elderly (13.8–21.9 years old) NHP models in both one- and two-dose regimens [29]. The study found no significant difference of immune response between adult and aged NHP’s, and as expected, the two-dose regimen elicited a greater immune response than a single dose in both groups. A different study compared the immunogenic properties of SARS-CoV-2 spike ectodomain, i.e., S1 with RBD and S2 with fusion domain in aged (15 months old) and young (6–8 weeks old) adult mice [30]. The authors found that higher doses of the vaccines and the addition of adjuvants were needed in order to induce SARS-CoV-2 spike-specific IgG antibodies, antibody isotypes, neutralizing antibodies, and T-cells; thus, this suggests that either a higher or multi-dose vaccination may be important considerations when vaccinating populations at an advanced age. Experiments in older animals (22 months old) also showed decreased antibody responses and a booster was required to obtain robust antibody responses [31]. Our AAV-based COVID-19 candidate is of interest in this realm as our studies were performed on 24-month-old mice, and some of the evaluated AAVCOVID candidates were able to induce an immune response in these aged models, even without the use of adjuvants. A study looking into the immunogenicity of the mRNA-1273 COVID vaccine in older adults found that both 25 µg and 100 µg doses were effective in eliciting an immune response in older adults, and that the 100 µg dose was more immunogenic [20]. In our study, we found that while responses were reduced in 2-year-old mice as compared to younger mice of the same strain, it remains unclear how these models predict the immunogenicity in older humans. Still, it was interesting that AC1 potency was qualitatively higher in these models compared to that of AC3.

Overall, AAVCOVID is a vaccine candidate for SARS-CoV-2 that can induce a robust immune response in animal models following a single injection. Specifically, AC1 enables the induction of potent humoral immune response in mouse models of age and obesity, which are attributes that are associated with increased vulnerability to COVID-19 in humans [28]. It was found that there was no significant difference in immune response between lean and DIO murine models, and the immune induction of AAVCOVID was still robust in aging models, though reduced. Of importance, we found that the low dose of the AC3 condition was less effective in inducing immunogenicity in aged mice than any other condition. A low AC3 dose is also the least effective candidate in the wild-type mice, suggesting that this condition may not be an effective vaccination methodology. Overall, responses were decreased in aged mice in comparison to younger mice of the same strain and condition. This type of animal study for COVID-19 has not yet been reported, and it is not clear how these models predict immunogenicity in older humans. The summation of these data suggests that AAVCOVID is a promising vaccine against SARS-CoV-2, but requires further examination against high-risk populations.

## 5. Limitations of the Study

The mouse models used in this study compared immune responses elicited by AAVCOVID vaccine candidates in obese and aged mice. However, mice are not considered a good model for protective efficacy assessment due to the lack of infection of some SARS-CoV-2 strains and poor resemblance with humans. Therefore, other models, such as hamsters or non-human primates, should be considered to assess protection from viral challenge. Additionally, the cellular response elicited by the vaccine could not be studied in aged mice due to their advance age.

## Data Availability

The data presented in this study are available on request from the corresponding author. The sequence of the vaccine candidates are available at GenBank (MW408785 and MW408786).

## Supplementary Materials

The following supporting information can be downloaded at: https://www.mdpi.com/article/10.3390/v14040820/s1. Figure S1: Weight of mice with a high fat diet (DIO) versus lean mice. Figure S2: Neutralization curves of individual mice lean or diet-induced obese (DIO) mice treated with AC1 and AC3 at two doses 28 days (upper row) and 56 days (lower row) after vaccination. Figure S3. Neutralization curves of individual young and aged mice treated with AC1 and AC3 at two doses 4 weeks (upper row) and 7–8 weeks (lower row) after vaccination.
