# Supplementary material for: Immunogenicity of an AAV-Based COVID-19 Vaccine in Murine Models of Obesity and Aging
Source: Viruses. 2022 Apr 15;14(4):820. doi: 10.3390/v14040820 (PMC9026898; doi:10.3390/v14040820)
Supplement: Supplementary file 1 [file viruses-14-00820-s001.zip › viruses-1651068-supplementary.pdf]

Figure S1

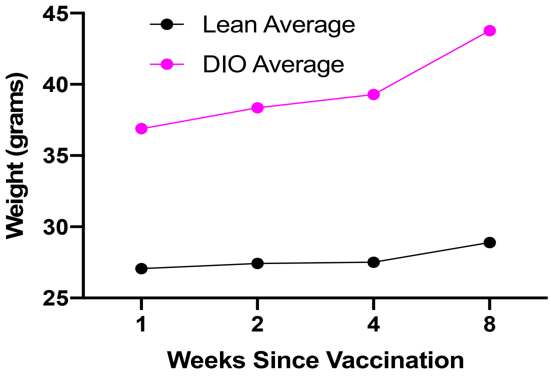

Figure S1. Weight of mice in high fat diet (DIO) versus lean mice. Data points represented as group average in grams. Dotted lines indicate lower limit of detection.

Figure S2

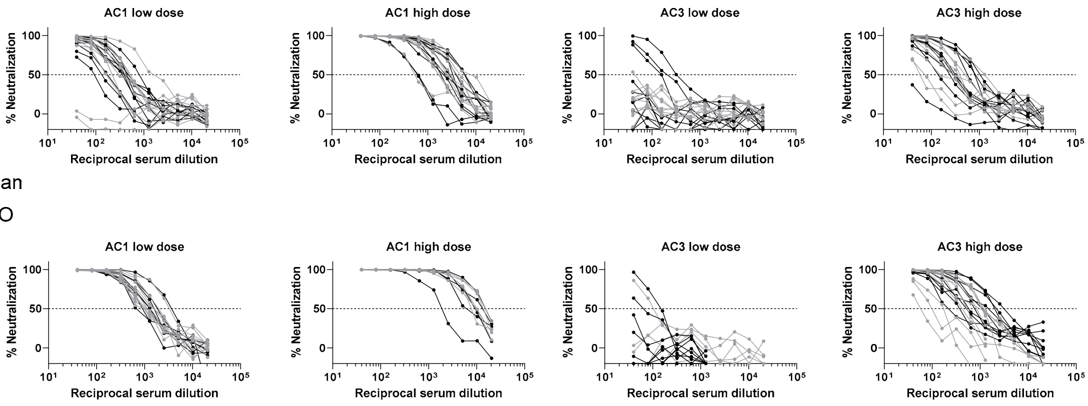

Figure S2. Neutralization curves of individual mice lean or diet-induced obese (DIO) mice treated with AC1 and AC3 at two doses 28 days (upper row) and 56 days (lower row) after vaccination.

Figure S3

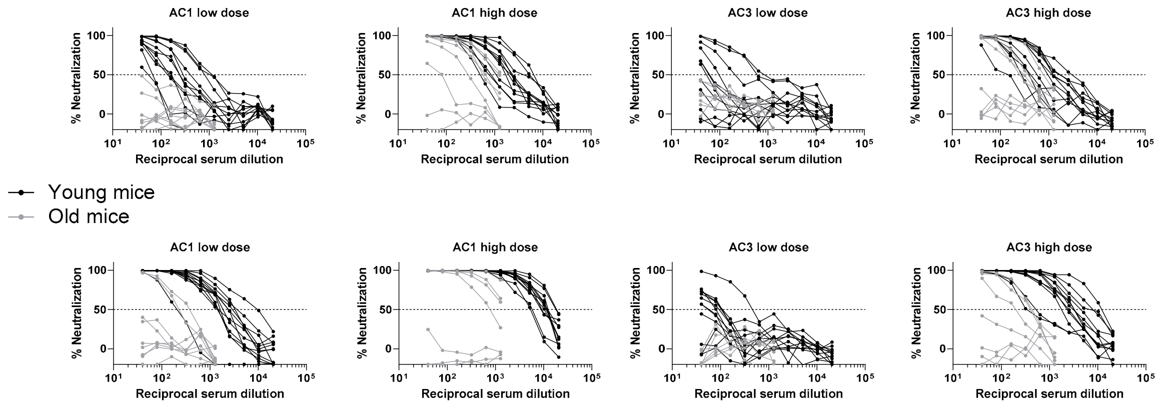

Figure S3. Neutralization curves of individual young and aged mice treated with AC1 and AC3 at two doses 4 weeks (upper row) and 7-8 weeks (lower row) after vaccination.
